# A Mitochondrial Autonomously Replicating Sequence from *Pichia pastoris* for Uniform High Level Recombinant Protein Production

**DOI:** 10.3389/fmicb.2017.00780

**Published:** 2017-05-02

**Authors:** Jan-Philipp Schwarzhans, Tobias Luttermann, Daniel Wibberg, Anika Winkler, Wolfgang Hübner, Thomas Huser, Jörn Kalinowski, Karl Friehs

**Affiliations:** ^1^Fermentation Engineering, Faculty of Technology, Bielefeld UniversityBielefeld, Germany; ^2^Microbial Genomics and Biotechnology, Center for Biotechnology (CeBiTec), Bielefeld UniversityBielefeld, Germany; ^3^Genome Research of Industrial Microorganisms, CeBiTec, Bielefeld UniversityBielefeld, Germany; ^4^Biomolecular Photonics, Faculty of Physics, Bielefeld UniversityBielefeld, Germany

**Keywords:** episomal vectors, mitochondrial DNA migration, non-homologous end joining, recombinant protein production, autonomously replicating sequence consensus sequence, *Pichia pastoris*, *Saccharomyces cerevisiae*, *Komagatella phaffii*

## Abstract

*Pichia pastoris* is a non-conventional methylotrophic yeast that is widely used for recombinant protein production, typically by stably integrating the target gene into the genome as part of an expression cassette. However, the comparatively high clonal variability associated with this approach usually necessitates a time intense screening step in order to find strains with the desired productivity. Some of the factors causing this clonal variability can be overcome using episomal vectors containing an autonomously replicating sequence (ARS). Here, we report on the discovery, characterization, and application of a fragment of mitochondrial DNA from *P. pastoris* for use as an ARS. First encountered as an off-target event in an experiment aiming for genomic integration, the newly created circular plasmid named “pMito” consists of the expression cassette and a fragment of mitochondrial DNA. Multiple matches to known ARS consensus sequence motifs, but no exact match to known chromosomal ARS from *P. pastoris* were detected on the fragment, indicating the presence of a novel ARS element. Different variants of pMito were successfully used for transformation and their productivity characteristics were assayed. All analyzed clones displayed a highly uniform expression level, exceeding by up to fourfold that of a reference with a single copy integrated in its genome. Expressed GFP could be localized exclusively to the cytoplasm via super-resolution fluorescence microscopy, indicating that pMito is present in the nucleus. While expression levels were homogenous among pMito clones, an apparent upper limit of expression was visible that could not be explained based on the gene dosage. Further investigation is necessary to fully understand the bottle-neck hindering this and other ARS vectors in *P. pastoris* from reaching their full capability. Lastly, we could demonstrate that the mitochondrial ARS from *P. pastoris* is also suitable for episomal vector transformation in *Saccharomyces cerevisiae*, widening the potential for biotechnological application. pMito displayed strong potential to reduce clonal variability in experiments targeting recombinant protein production. These findings also showcase the as of yet largely untapped potential of mitochondrial ARS from different yeasts for biotechnological applications.

## Introduction

Since its discovery in the 1970s and the development of first molecular genetic tools in the 1980s, the non-conventional yeast *Pichia pastoris* has become a widely used host for recombinant protein production (Cregg et al., 1985; Ellis et al., 1985). Although recent research resulted in the reclassification of the most commonly used *P. pastoris* strains into *Komagatella phaffii* or *K. pastoris* (Kurtzman, 2005, 2009), the old name remains the popular choice for describing these organisms. The capability for high level protein production and secretion, post-translational modifications and ease of cultivation allowed the successful expression of a multitude of proteins, ranging from technical enzymes like phytase to biopharmaceuticals like the kallikrein inhibitor Kalbitor^®^ (Ahmad et al., 2014; Bill, 2014). Consequently, much effort has been put into better understanding the genomic (Sturmberger et al., 2016; Valli et al., 2016), transcriptomic (Love et al., 2016) and metabolic (Rußmayer et al., 2015; Irani et al., 2016) properties of this host organism, in order to improve recombinant protein yields. In the last years, many studies provided novel regulatory elements, especially promoters for recombinant protein production in *P. pastoris* (Qin et al., 2011; Prielhofer et al., 2013; Vogl et al., 2016). The best studied and most commonly applied promoter in *P. pastoris* is the alcohol oxidase 1 (*AOX1*) promoter (Vogl and Glieder, 2013). It offers tight regulation, exceptionally high expression levels and can be induced with methanol.

Typically, the expression cassette containing the gene of interest is integrated into the chromosome via homologous recombination, enabling high genetic stability and, if desired, a simultaneous knock-out at the targeted locus (Klinner and Schäfer, 2004; Ahmad et al., 2014). Multicopy clones can be generated with different strategies to increase gene dosage and productivity (Marx et al., 2009; Aw and Polizzi, 2013, 2016). However, high copy numbers can also lead to genetic instability and the loss of expression cassettes during cultivation (Zhu et al., 2009). Furthermore, off-target integrations due to non-homologous end joining (NHEJ) events, increased cell stress caused by high gene dosage, and other as of yet not fully understood factors can lead to a heterogeneous productivity landscape in strains transformed with an integrative expression cassette (Clare et al., 1991; Hohenblum et al., 2004; Mattanovich et al., 2004; Cámara et al., 2016; Schwarzhans et al., 2016a). Our previous study revealed non-canonical NHEJ mediated integration events including reintegration of the knock-out target on a different chromosome and co-integration of *Escherichia coli* plasmid host DNA (Schwarzhans et al., 2016b). In contrast to *Saccharomyces cerevisiae*, the NHEJ pathway dominates over homologous recombination in *P. pastoris*, similar to many other yeasts and higher eukaryotes (Guirouilh-Barbat et al., 2004; Daley et al., 2005; Meyer et al., 2007; Näätsaari et al., 2012).

The classic way of overcoming some of the disadvantages associated with integrative expression cassettes, in particular genetic perturbance, is the use of episomal vectors. Since the discovery of the 2 μm plasmid in *S. cerevisiae*, several plasmids containing autonomously replicating sequences (ARS) for plasmid propagation have been developed (Futcher, 1988; Christianson et al., 1992; Newlon and Theis, 1993). Further research led to the detection of ARS in other yeasts like *Kluyveromyces lactis*, *Schizosaccharomyces pombe*, and *P. pastoris* (Peng et al., 2015). In some cases, episomal ARS vectors and integration of the expression cassette are combined into one strategy. For example, the classic *P. pastoris* ARS *PARS1* has been used both for episomal circular vectors as well as for *in vivo* amplification of linear plasmids prior to genomic integration (Lee et al., 2005; Madsen et al., 2016). A genome-wide study of *P. pastoris* GS115 led to the discovery of a multitude of (putative) ARS elements on the chromosomal DNA (Liachko et al., 2014). However, this analysis excluded the mitochondrial genome. Recently, a novel ARS originating from *K. lactis* and capable of plasmid propagation in a wide range of (non-) conventional yeasts was shown to be a promising candidate for episomal recombinant protein expression in *P. pastoris* (Liachko and Dunham, 2014; Camattari et al., 2016).

In many eukaryotes, ranging from yeasts to higher plants, animals and humans, the occurrence of mitochondrial DNA (mtDNA) on chromosomal DNA has been observed (Blanchard and Schmidt, 1996; Ricchetti et al., 2004; Hazkani-Covo et al., 2010). While the exact mechanism of mtDNA migration from the mitochondrion to the nucleus is not yet fully understood, the data suggests that the number of mtDNA integrations correlates with the genome size (Hazkani-Covo et al., 2010). It could also be shown that the integration of mtDNA into chromosomal DNA relies on the NHEJ repair of double-strand breaks (DSBs) (Ricchetti et al., 1999). In extreme cases, mtDNA integration can lead to genetic diseases (Turner et al., 2003), but most integrations have been localized to intergenic, intron or telomeric regions (Bernatzky et al., 1989; Louis and Haber, 1991; Noutsos et al., 2007). Nuclear mtDNA elements have been well-studied in *S. cerevisiae*, revealing their localizations, frequencies and properties (Sacerdot et al., 2008; Chatre and Ricchetti, 2011; Dujon, 2012). Some mtDNA elements from *S. cerevisiae* exhibit ARS activity (Gunge, 1983; Hyman et al., 1983; Delouya and Nobrega, 1991). Furthermore, in a study by Schiestl et al. (1993) aimed to induce non-homologous integrations in *S. cerevisiae*, an *in vivo* ligation of transformed DNA and mtDNA leading to the creation of a replicating plasmid was detected. So far, no data has been published on mtDNA migration, on ARS elements of mtDNA or the application of such elements from a biotechnological perspective in *P. pastoris*.

Here, we report on the discovery of a novel mtDNA ARS in *P. pastoris* and its application for episomal plasmid propagation. The ARS was first found by using genome sequencing in an experiment employing an integrative expression cassette. After validation of the presence of a circular plasmid in the affected strain, the ARS vector was assessed for its productivity characteristics. The characterization experiments indicate a uniform and high level recombinant protein production and favorable cellular localization of the product, confirmed by super-resolution fluorescence microscopy. Lastly, we could demonstrate that the mtDNA ARS can also be used for episomal transformation in *S. cerevisiae*.

## Results

### Discovery of pMito

**FIGURE 1:**
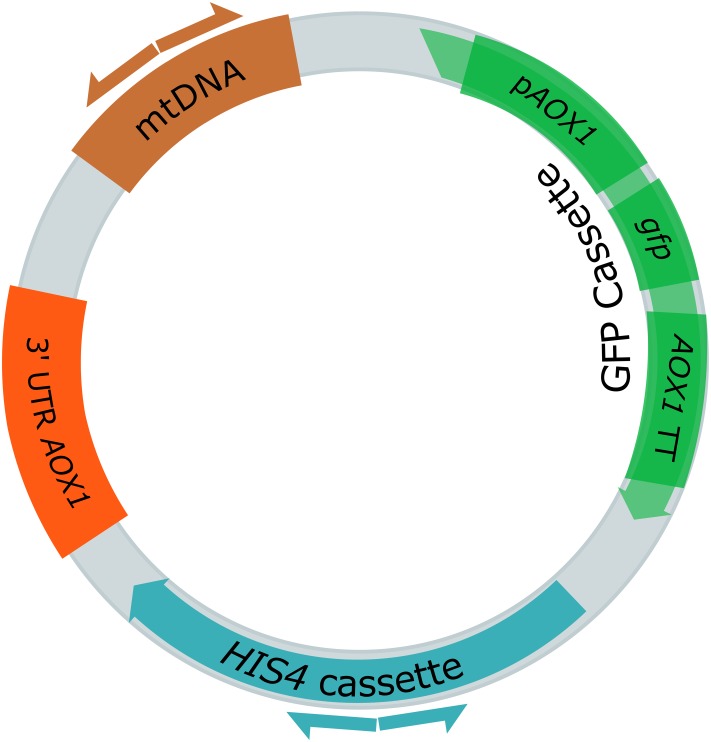
**Plasmid map of the 7.3 kb pMito, as discovered in *Pichia pastoris* JPS664.** The displayed features include the GFP cassette, consisting of the *AOX1* promoter (p*AOX1*), *gfp* gene and *AOX1* terminator (*AOX1* TT), the *HIS4* cassette as selection marker, the 3′ UTR *AOX1* (in original vector for homologous integration purposes) and a fragment of mtDNA. Direction and binding sites of primers pairs used for PCR assays are shown as differently colored half arrows: pMito_Circ-mtDNA-FW/RV (brown) and pMito_Circ-HIS4-FW/RV (blue).

In our previous study, we analyzed a library of 845 *P. pastoris* clones transformed with an integrative GFP expression cassette for their productivity characteristics (Schwarzhans et al., 2016a). Based on the assayed features, interesting clones were selected for genome sequencing. Some strains were selected, because they displayed GFP expression levels that far exceeded the one predicted based on their gene copy number (GCN). One of these was strain JPS664 (EMBL FBUC01000000). With a GCN = 1 a normalized GFP expression level of ca. 1 was to be expected. However, a normalized expression level of 2.4 ± 0.3 was found. Consequently, JPS664 was selected for genome sequencing. After sequencing, it was revealed that JPS664 did not contain an expression cassette in its chromosome. Rather, a ligation of the GFP cassette to a 1.4 kb fragment of mitochondrial DNA was found. As a result of the fusion, a circular 7.3 kb plasmid was formed that was named “pMito” (**Figure 1**, EMBL LT724168). Due to the plasmid character of pMito, an ARS was suspected to be encoded on the mtDNA fragment. The mitochondrial DNA shows 100% identity to the bases 27,552–28,993 of the mitochondrial genome of *P. pastoris* CBS7435 containing a fragment of the *COX1* (cytochrome c oxidase I) gene. The last 73 bp of the second *COX1* exon as well as 1369 bp of the second *COX1* intron were detected on the segment. The combination of a novel mtDNA *in vivo* ligation to an expression cassette in *P. pastoris*, potential ARS activity, and the apparently high suitability of the plasmid for recombinant protein production prompted us to conduct further experiments on pMito.

### Validation of Plasmid Character of pMito

**FIGURE 2:**
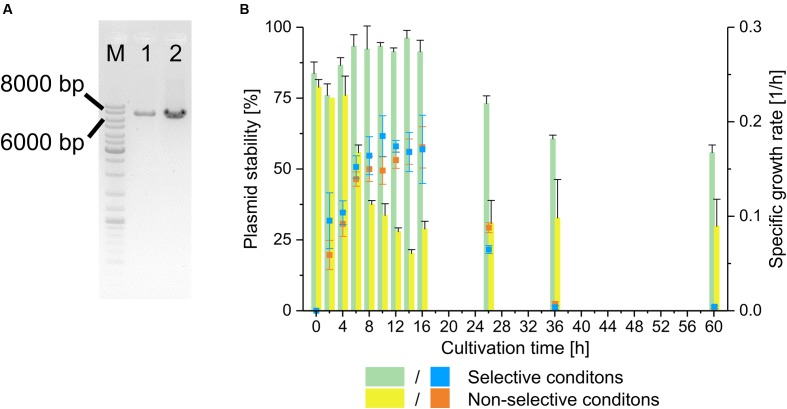
**Validation of pMito circular plasmid characteristics.** **(A)** PCR assay for presence of circular pMito in JPS664. Lanes: (M) Marker (1) Primer pair pMito_Circ-HIS4-FW/RV (2) Primer pair pMito_Circ-mtDNA-FW/RV. Relevant band sizes have been highlighted. **(B)** Plasmid stability (columns) and specific growth rate (symbols) of pMito in JPS664, grown under selective and non-selective conditions. Error bars represent the standard deviation, with *n* = 2.

First, the plasmid character of pMito had to be validated in order to ensure that it was not an artifact based on genome sequencing and assembly. To this end, a PCR assay with two sets of directly adjacent, diverging primer pairs was designed. This way, a PCR product of the same size as the predicted pMito would validate its circular structure. One primer pair binds in the *HIS4* region of pMito and the other in the mtDNA fragment. In **Figure 2A** the PCR assay visibly indicates the presence of a full length circular plasmid as shown in **Figure 1**. In addition, the plasmid stability of pMito was investigated (**Figure 2B**). Under selective conditions pMito is well-maintained at up to 96.2 ± 2.7%, while under non-selective conditions the plasmid stability was lowest at 20.2 ± 1.4%. In combination with the specific growth rate, a growth associated loss and increase of pMito content is apparent, respectively. On the one hand, pMito content rose, while cells grew in selective media and stagnated or fell when they reached the stationary phase, indicating that cells containing pMito lost their growth advantage over plasmid-free cells. On the other hand, cells grown in non-selective media quickly lost pMito during the exponential growth phase and exhibited constant plasmid contents in the stationary phase, indicating a growth advantage of plasmid-free cells. Taken together, the plasmid stability and PCR assay clearly validated the circular plasmid character of pMito.

### *In Silico* Analysis of pMito

**FIGURE 3:**
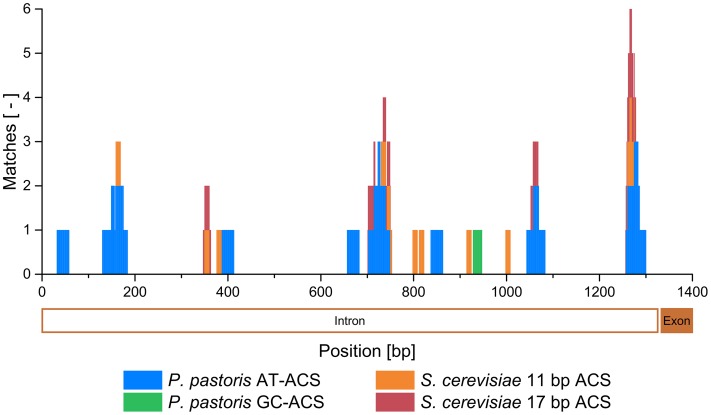
**Cumulative matches of the FIMO scan for *P. pastoris* AT- and GC-ACS, as well as *S. cerevisiae* 11 and 17 bp ACS motifs mapped to the mtDNA fragment of pMito.** The corresponding second exon and second intron region of the *COX1* gene are shown below the x-axis in an orientation from right to left.

It was therefore highly likely, that the mtDNA fragment in pMito conferred ARS activity. Using the Find Individual Motif Occurrences (FIMO) tool (Grant et al., 2011), the mtDNA fragment was scanned for known ARS consensus sequence (ACS) motifs. Both AT- and GC-rich ACS motifs from *P. pastoris* as well as the 11 and the 17 bp ACS motifs of *S. cerevisiae* were included (Newlon and Theis, 1993; Theis and Newlon, 1997; Liachko et al., 2014). Despite the low GC-content (22%) of the mtDNA fragment, one putative GC-ACS was found in addition to several AT-ACS and *S. cerevisiae* ACS with a total of 33 matches (**Table 1**). A more detailed description of the FIMO matches can be found in Supplementary Table S1. By mapping the FIMO matches to the sequence of the mtDNA fragment, multiple clusters of predicted ARS become visible (**Figure 3**). All putative ARS were found in the *COX1* intron on the mtDNA. Especially in the regions at 1256–1300 bp and 701–753 bp matching sequences were found, with up to 6 and 4 cumulative matches, respectively. Here, similar sequences to the *P. pastoris* AT-ACS, *S. cerevisiae* 11 bp ACS and *S. cerevisiae* 17 bp ACS were detected. The accumulation of different types of ACS indicates a high likelihood of actual ARS functionality in these regions. Additionally, between 2 and 3 cumulative matches can be found in the regions of 130–184, 347–363, and 1043–1083 bp. In these regions as well, ARS activity could be present. Although *P. pastoris* ACS motif matches were found, no region of the mtDNA fragment could be directly aligned via BLASTn to the library of (putative) ARS sequences on the *P. pastoris* GS115 chromosomes (Liachko et al., 2014). This indicates that the ARS on pMito, while similar to the ARS found on the chromosomes, has its own distinguishable sequence. A preliminary FIMO scan of the mitochondrial genome of *P. pastoris* CBS7435 (GenBank: FR839632) identified 20 GC-ACS and over 500 AT-ACS motif occurrences (data not shown).

**Table 1:** Number of ACS motif matches found on the mtDNA fragment in pMito using FIMO.

Motif	Number of matches
*P. pastoris* AT-ACS	15
*P. pastoris* GC-ACS	1
*S. cerevisiae* 11 bp ACS	11
*S. cerevisiae* 17 bp ACS	6


The expression cassette portion of pMito, excluding the mtDNA fragment, was also scanned for ACS motif occurrences, as described above. In spite of being approximately four times as long as the mtDNA fragment, only five matches were found. They are detailed in Supplementary Table S2 and the mapping result is shown in Supplementary Figure S1. Interestingly, three of these matches accumulate within a 43 bp region of the *AOX1* terminator and two occur in the 3′ UTR *AOX1* element. Therefore, we conducted transformation experiments with different variants of the plasmid.

### Transformation of pMito Variants into *P. pastoris*

**FIGURE 4:**
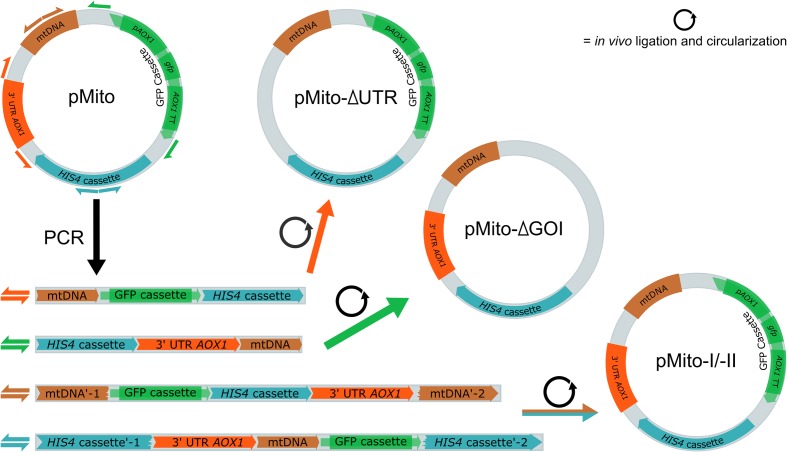
**Schematic overview of the construction and *in vivo* assembly of four different pMito variants used for transformation.** Direction and primer binding sites of primers pairs used for construction are shown as differently colored half arrows: pMito_Circ-mtDNA-FW/RV (brown), pMito_Circ-HIS4-FW/RV (blue), pMito_No_GOI-FW/RV (green) and pMito_No_UTR-FW/RV (orange). In the first step, the variants were amplified as linear DNA via PCR. After successful transformation into *P. pastoris*, linear DNA was *in vivo* ligated into circular plasmids.

Using the original pMito from JPS664 as template, four different variants were created via PCR (**Figure 4**). They differed in two aspects. Firstly, the constructs pMito-I and pMito-II encompass the complete plasmid, while pMito-ΔUTR (untranslated region) omits the 3′ UTR *AOX1* region and pMito-ΔGOI (gene of interest) does not contain the *AOX1* promoter, *gfp* gene and *AOX1* terminator. Secondly, different loci were used for linearization. pMito-ΔUTR and pMito-ΔGOI were linearized by removing the aforementioned segments. pMito-I was linearized inside the mtDNA fragment and pMito-II inside the *HIS4* gene. In accordance with our previous recommendations, *P. pastoris* was transformed with linear, PCR amplified DNA (Schwarzhans et al., 2016b). This method also offered the advantage to assay *P. pastoris* capability for *in vivo* circularization of the transformed DNA. In the case of pMito-II, only a successfully circularized plasmid could bestow histidine prototrophy, since the *HIS4* gene was split onto the distal ends of the transformed, linear DNA.

**FIGURE 5:**
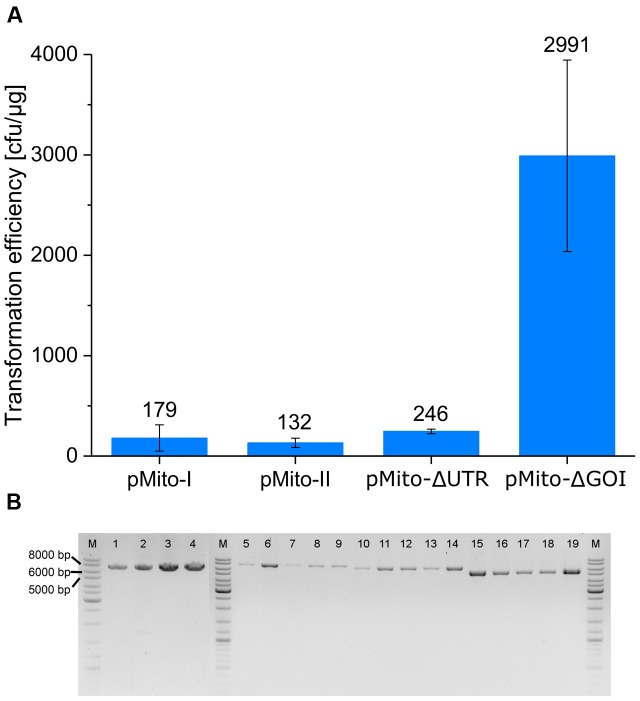
**(A)** *P. pastoris* transformation efficiencies of the four pMito variants. Error bars represent the standard deviation, with *n* = 3. **(B)** PCR assay for circular plasmids in different pMito clones, using the primer pair pMito_Circ-mtDNA-FW/RV. Lanes: (M) Marker (1–4) pMito-I strains (5–9) pMito-II strains (10–14) pMito-ΔUTR strains (15–19) pMito-ΔGOI strains. Relevant band sizes have been highlighted.

All four constructs produced transformants (**Figure 5A**). Using the PCR assay described in the previous chapter, the presence of full-length circular plasmids could be confirmed in strains of all four constructs (**Figure 5B**). The successful transformation of pMito-II confirmed that *P. pastoris* was capable of *in vivo* ligating the linear DNA into circular plasmids. Depending on the aim of an experiment, this ability can be used, e.g., for DNA assembly purposes. Transformation of pMito-I, pMito-II, and pMito-ΔUTR resulted in comparable efficiencies, with an average of about 190 colony forming units (cfu)/μg. In contrast, an efficiency more than 10-fold higher at ca. 3000 cfu/μg was encountered using pMito-ΔGOI.

### Productivity Characterization of pMito-I Strains

**FIGURE 6:**
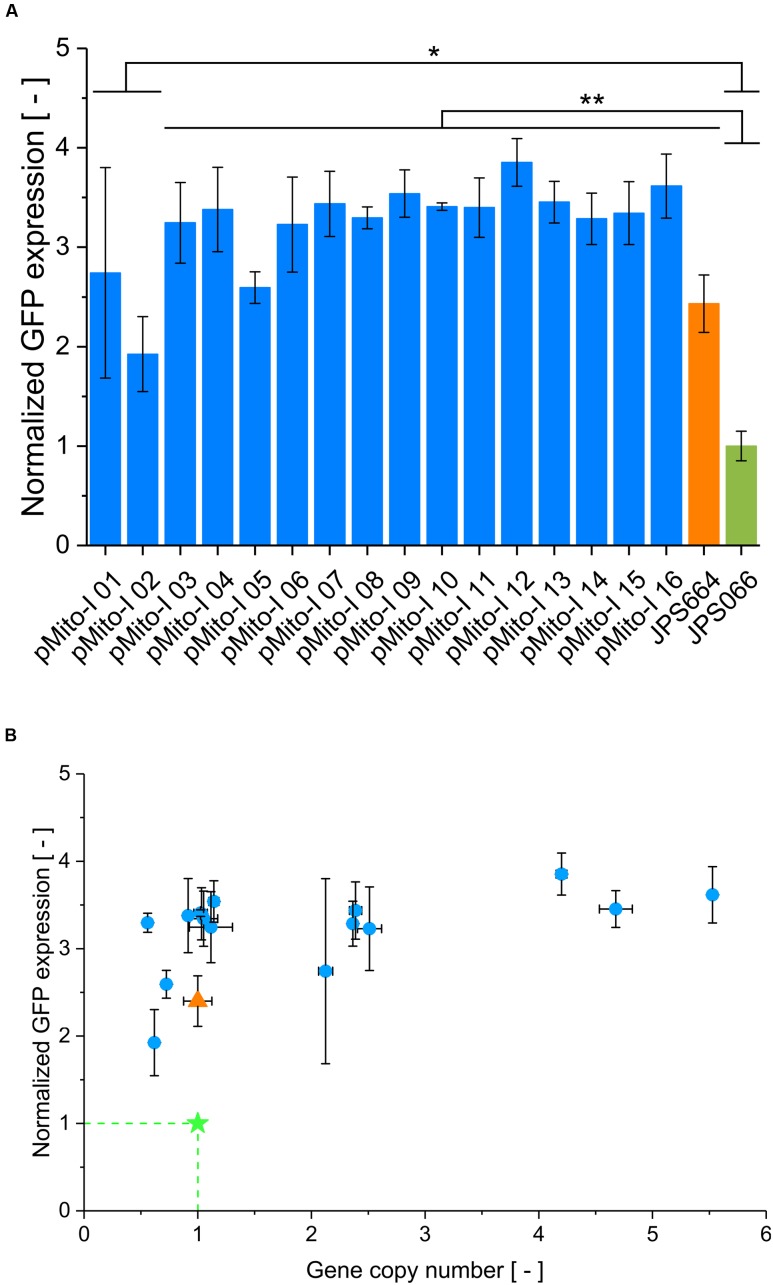
**Productivity characterization of 16 pMito-I strains** **(A)** Normalized expression level of 16 pMito-I strains, JPS664 and JPS066. Applying the Student’s *t*-test (two-sided), the significance of divergence from JPS066 was assayed. ^∗^*p*-value < 0.05 and ^∗∗^*p*-value < 0.01. Error bars represent the standard deviation, with *n* = 3. **(B)** Normalized expression level and GCN of the 16 pMito-I strains (blue dots). JPS066 is shown as green star and JPS664 as orange triangle. Error bars indicate the standard deviation, with *n* = 3.

Since the original strain JPS664 in which pMito was found exhibited favorable productivity characteristics, it was of interest to investigate if these properties could be replicated. To this end, 16 clones transformed with pMito-I were randomly selected and submitted to further analysis. The productivity of the selected clones was assayed in relation to the original JPS664 and the reference strain JPS066, which contained a single expression cassette integrated into the *AOX1* locus on the genome (Schwarzhans et al., 2016a). All selected pMito-I clones produced high amounts of GFP, markedly exceeding the reference strain at least two- and up to fourfold (**Figure 6A**). Applying the Student’s *t*-test (two-sided), it was determined that all pMito clones, and the original strain JPS664, produced GFP at significantly higher levels than JPS066, with the vast majority of pMito-I strains scoring a *p*-value < 0.01. A highly uniform expression level of pMito-I strains is visible, with all but one clone (pMito-I 2) exhibiting normalized expression levels in the range of 2.5–3.9. It seemed, that from the normalized expression levels an upper limit of GFP productivity can be deduced. To investigate whether the gene dosage could explain this behavior all clones were subjected to GCN analysis. As can be seen in **Figure 6B**, expression level and GCN did not correlate. While GCN values between 0.6 and 5.5 were encountered, the gene dosage of a strain had apparently no impact on the expression level.

### Intracellular Localization of Expressed GFP

**FIGURE 7:**
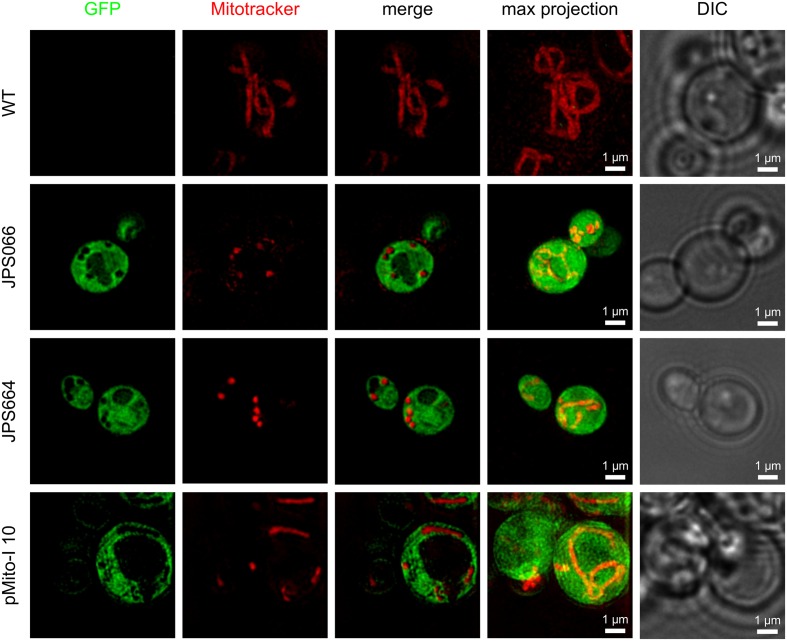
**Super-resolution fluorescence microscopy images of living *P. pastoris* cells expressing GFP and stained with MitoTracker^®^ Red CM-H_2_XRos.** Images were acquired in the 3D SR-SIM mode. The *P. pastoris* CBS7435 wild type (WT), strains JPS066 and JPS664, as well as clone pMito-I 10 were analyzed. For all strains, a single z plane image of the GFP and MitoTracker signal are shown separately, merged and then as maximum intensity projection of the recorded entire cell z-stack (4–8 μm depth). In the last column a corresponding DIC image is displayed.

Not only the expression level but also the localization of the product is of interest. For correct post-translational modification and secretion of the recombinant protein, it must first be present in the cytoplasm, so it can translocate to the endoplasmic reticulum and Golgi apparatus. A post-translational translocation of GFP to the mitochondria was unlikely due to the absence of a corresponding signal sequence. However, it was possible that copies of pMito were present in the mitochondria, especially for JPS664. Here, the exact origin of pMito was uncertain and could result in GFP accumulation in the mitochondria, complicating product capture. Living cells of the *P. pastoris* CBS7435 wild type (WT), JPS664, JPS066 and strain pMito-I 10 were analyzed via super-resolution fluorescence microscopy. In all GFP expressing strains the recombinant protein was present in the entire cytoplasm (**Figure 7**). Two major compartments absent of GFP fluorescence are distinguishable. They are likely the peroxisome and the mitochondria. This and more findings are analyzed in more detail in Section “Discussion”.

### mtDNA ARS from *P. pastoris* in *S. cerevisiae*

**FIGURE 8:**
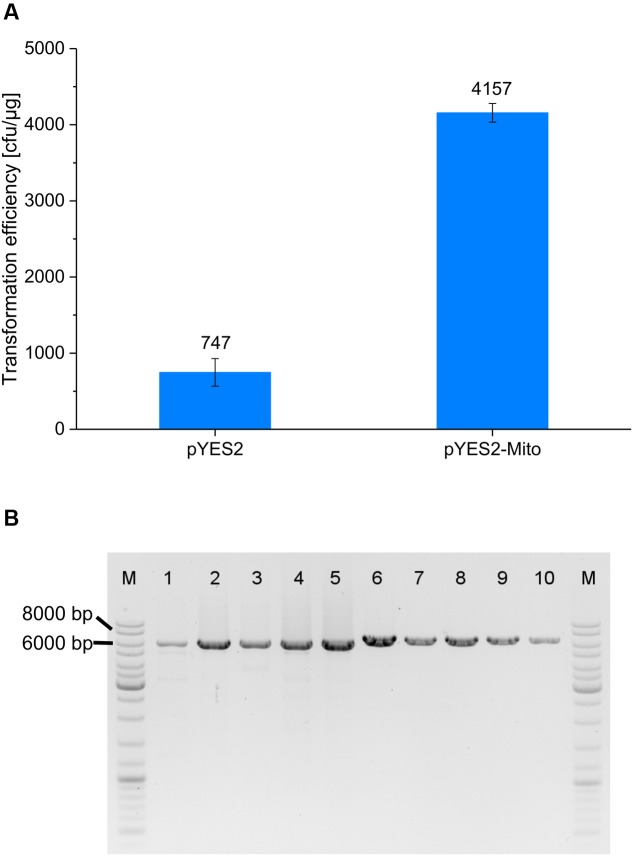
**(A)** *S. cerevisiae* transformation efficiencies of pYES2 (5.9 kb) and pYES2-Mito (6.4 kb). Error bars represent the standard deviation, with *n* = 3. **(B)** PCR assay for circular plasmids in pYES2 and pYES2-Mito clones. Lanes: (M) Marker (1–5) PCR product of five pYES2 strains, using the primer pair pYES2_Circ-FW/RV (6–10) PCR product of five pYES2-Mito strains, applying the primer pair pMito_Circ-mtDNA-FW/RV. The size of relevant bands has been highlighted.

As previously detailed, ACS motifs from *S. cerevisiae* could be matched to the mtDNA segment of pMito. Therefore, we were interested to see if pMito could also be used as an episomal vector in *S. cerevisiae*. A variant of the plasmid pYES2 was constructed, replacing the 2 μm origin with the mtDNA fragment of pMito, resulting in pYES2-Mito. For the purpose of better comparison with the *Pichia* experiments, *S. cerevisiae* was transformed with linear, PCR amplified constructs. Both constructs facilitated creation of uracil prototroph *S. cerevisiae* clones (**Figure 8A**) and the presence of circular plasmids was confirmed via PCR (**Figure 8B**). Surprisingly, the transformation efficiency of pYES2-Mito was almost sixfold higher as for pYES2.

## Discussion

During an experiment targeting the genomic integration of a GFP expression cassette in *P. pastoris* the random creation of the circular plasmid pMito, consisting of the expression cassette and a 1.4 kb fragment of mtDNA, was discovered. Due to the good productivity characteristics of the affected strain the plasmid was further investigated.

It is likely that pMito is the result of NHEJ mediated DSB repair, consistent with previous reports on the involvement of mtDNA in DSB repair (Ricchetti et al., 1999). So far no involvement of mtDNA in DSB repair or the formation of replicating plasmids due to its involvement have been reported for *P. pastoris*. Potentially, *P. pastoris* ligated the transformed, linear expression cassette with the fragment of mtDNA in order to “repair” the expression cassette. This untargeted incident is reminiscent of other non-canonical NHEJ mediated integration events in *P. pastoris* we have reported on previously, e.g., the *in vivo* ligation of *E. coli* DNA fragments to the expression cassette (Schwarzhans et al., 2016b). The presence of mtDNA in the nucleus has been reported before, although the exact mechanism of the mtDNA migration is unclear (Hazkani-Covo et al., 2010). However, similar events have been observed in *S. cerevisiae*. For this model yeast it is known that its mtDNA is rich in ARS and can migrate to the chromosomes (Hyman et al., 1983; Sacerdot et al., 2008). Furthermore, it has been shown that under conditions that favor NHEJ, *in vivo* ligation of transformed DNA to mtDNA can occur, forming replicating plasmids (Schiestl et al., 1993).

Applying two sets of diverging primer pairs the circular structure of pMito was confirmed via PCR. During the plasmid stability assay it is possible that L-histidine was released into the medium by prototrophic strains, making it easier for plasmid-free cells to propagate, even under selective conditions. Similar observations have been made in experiments with other auxotrophic yeast strains (Pronk, 2002). It seems likely that if nutrient limitation could be avoided and cells had continued to grow, the plasmid content would have dropped further under non-selective conditions.

Applying the FIMO tool and known ACS motifs from *P. pastoris* and *S. cerevisiae*, multiple matches for potential ARS sites were found on the mtDNA fragment. Besides the mtDNA segment, five additional ACS motif matches were found on the *AOX1* terminator associated regions of pMito. Some *S. cerevisiae* terminator regions are associated with ARS activity (Chen et al., 1996). However, Vogl et al. (2016) tested various *P. pastoris* terminators for ARS functionality and determined that the *AOX1* terminator has no ARS activity. Especially in the regions 701–753 and 1256–1300 bp of the mtDNA segment, a clustering of putative ARS sequences was observed, making them promising candidates for further experiments. For instance, they could serve as starting point to reduce the size of the mtDNA fragment needed for ARS activity. Interestingly, none of the predicted ARS matched the ones found on the chromosomes of *P. pastoris* (Liachko et al., 2014), indicating the presence of a novel ARS sequence on pMito. Over 500 additional ACS matches were found in a preliminary FIMO scan of the mitochondrial genome of *P. pastoris* CBS7435, suggesting it is tightly packed with potential ARS elements, similar to *S. cerevisiae* (Hyman et al., 1983).

We noticed a more than 10-fold increase in transformation efficiency, if using the pMito-ΔGOI construct compared to pMito-I, -II, and pMito-ΔUTR. Two main factors might have contributed to this increase in transformation efficiency. Firstly, pMito-ΔGOI is the shortest of the four constructs with 5.3 kb (pMito-I and -II: 7.3 kb, pMito-ΔUTR: 6.6 kb). The smaller size could have eased entry of the foreign DNA into the cell or nucleus and made circularization of the linear DNA via *in vivo* ligation more efficient, e.g., by containing less secondary structures than longer variants. To this end, the above mentioned clustering of putative ARS on the mtDNA segment could serve as starting point for constructing smaller variants with higher transformation efficiency. Secondly, by omitting the GOI region three ACS motif matches in the *AOX1* terminator are eliminated. Although, it has been shown that the *AOX1* terminator possesses no ARS activity, the matching sequences could still facilitate interaction with the origin recognition complex (ORC), needed for DNA replication (Bell and Stillman, 1992). Therefore, in constructs containing the *AOX1* terminator potentially less ORC was free to initiate DNA replication at one of the ARS sites of the mtDNA of pMito. Especially, directly after transformation, when cell survival is most dependent on the histidine prototrophy conferred by the plasmid encoded *HIS4*, a delayed or inhibited replication of pMito could lead to cell death. This interference would also cause lower plasmid stability, meaning a lower frequency of daughter cells containing pMito. In consequence, even a successful transformation might have resulted in no cell growth or a markedly delayed cell growth. A similar, albeit much less pronounced, trend is visible when comparing the slightly increased transformation efficiency of pMito-ΔUTR to pMito-I and -II. Here, two ACS matches contained in the 3′ *AOX1* UTR are removed and the vector size is slightly reduced compared to the full length pMito.

In comparison to recently published results for ARS based protein production in *P. pastoris*, pMito surpasses the two tested chromosomal ARS vectors from *P. pastoris* in productivity and is approximately on par with the ARS vector from *K. lactis* (Camattari et al., 2016). Normalized expression levels were up to fourfold higher than the reference strain JPS066 and uniform among 16 pMito-I clones and the original JPS664 strain. Typically, transformation of *P. pastoris* results in strains with a wide range of productivities. For example, Clare et al. (1991) observed up to 30-fold differences in product titer, while Cámara et al. (2016) and our previous study (Schwarzhans et al., 2016a) reported on clonal variabilities ranging from non-producing to exceedingly high producing strains in transformants from one experiment. The high uniformity of pMito strains could help streamline screening procedures, which typically are time and work intense steps necessary in order to find strains with the desired productivity (Looser et al., 2015).

However, a seemingly gene dosage independent upper limit of GFP productivity was apparent. A similar phenomenon has been encountered by Camattari et al. (2016) in their characterization of different *P. pastoris* strains expressing blue fluorescent protein (BFP) from an ARS-based episomal vector. As in their case, recombinant protein expression might have been affected by post-translational or epigenetic factors (Love et al., 2010). It has to be noted that in our case GFP was expressed in the cytoplasm. However, the occurrence of highly similar phenomena in two studies working with episomal vectors in *P. pastoris* could point to a common origin. Especially, the potential connection to ARS based vectors would need additional investigation for clarifying the root cause, e.g., whether *Pichia* can distinguish between episomal and chromosomal expression and direct its resources accordingly. Further research is needed to fully understand the bottle-neck that might be preventing ARS based vectors from reaching their theoretical potential in *P. pastoris*.

It is known, that yeast mitochondria are capable of facilitating protein synthesis within the organelle (Herrmann et al., 2013). We wanted to ensure, that recombinant protein produced with pMito localized to the cytoplasm, enabling biotechnological application. To this end, we applied super-resolution fluorescence microscopy, which allows for the intracellular localization of fluorescent targets (Huang et al., 2009). This study marks the first reported implementation of this technique for analysis of *P. pastoris*. We could localize expressed GFP to the cytoplasm and identified two major compartments devoid of GFP. Firstly, what is assumed to be peroxisomes which have increased to multiple times their normal size due to the methanol induction (Johnson et al., 1999). Secondly, via a combination of the GFP and MitoTracker signal it becomes apparent, that the mitochondria contained no GFP. Best visible in the maximum intensity projection images, a tubular organization of the mitochondria can be clearly seen. This projection uses the entire recorded z-stack of the cell and thereby gives augmented information on the 3D spatial organization of fluorescence tagged targets. A similar structural organization was reported for *S. cerevisiae* mitochondria (Egner et al., 2002). Super-resolution fluorescence microscopy allowed for a more detailed insight into the spatial organization of mitochondria inside the living cell. This technique could therefore be of benefit for experiments studying intracellular localization of fluorescence-tagged targets in *P. pastoris*, where so far confocal microscopy has been used primarily (Heiss et al., 2015; Rueda et al., 2016). Cells were not fixed to avoid loss of the MitoTracker signal, therefore the “rings” visible in the DIC images are suspected to be an artifact caused by the living cells.

Lastly, the ARS activity in *S. cerevisiae* of the mtDNA segment from pMito was demonstrated via the plasmid pYES2-Mito. Higher transformation efficiencies were recorded for pYES2-Mito than for the original pYES2. This suggests that the ARS of pYES2-Mito is not only active in *S. cerevisiae*, but also easier to transform than the 2 μm based vector. However, it does not necessarily mean that pYES2-Mito outperforms pYES2 and other 2 μm based vectors in regards to recombinant protein productivity. 2 μm plasmids are known for their high copy number, enabling an increased gene dosage of the target gene (Christianson et al., 1992). It has to be noted, that the recombinant protein productivity in *S. cerevisiae* was not yet assayed but characterization of the applicative potential of pMito-derived vectors in this organism is subject to further studies. As it stands, the results clearly suggest the inter-genus capability of the ARS encoded on the mtDNA fragment of pMito.

In summary, the mtDNA fragment of pMito represents a promising candidate for ARS based recombinant protein production in *P. pastoris*, reducing clonal variability while providing increased expression levels. To fully gauge its production capabilities, the expression of additional (secreted) heterologous proteins via pMito is advisable. It could present itself as an alternative to integrative expression cassettes, especially when screening procedures are the limiting step. Combined with the high density of putative ARS on the whole mitochondrial genome of *P. pastoris*, a wealth of so far unused mtDNA ARS could benefit biotechnological and basic science approaches. Considering the relatively wide taxonomic distance of *P. pastoris* (order: Saccharomycetales; family: *Phaffomycetaceae*) and *S. cerevisiae* (order: Saccharomycetales; family: *Saccharomycetaceae*), the discovered ARS seems to be capable of a wider host range activity, akin to the *K. lactis* ARS “panARS,” discovered and optimized by Liachko and Dunham (2014). For panARS a very broad host range of 10 budding yeasts was shown, prompting us to explore a wider host range for pMito in further studies.

## Materials and Methods

### Microorganisms and Cultivation Conditions

All plasmids were constructed and propagated in *E. coli* Top 10 [Invitrogen, USA; genotype: F- *mcr*A Δ(*mrr-hsd*RMS*-mcr*BC) Φ80*lac*ZΔM15 Δ*lac*X74 *rec*A1 *ara*D139 Δ(*araleu*)7697 *gal*U *gal*K *rps*L (StrR) *end*A1 *nup*G]. *E. coli* cultivations were carried out in LB (Lysogeny Broth) medium supplemented with 100 μg/mL ampicillin. Experiments involving *P. pastoris* employed the WT strain CBS7435 (identical to NRRL Y-11430 and ATCC76273), obtained from the Spanish Type Culture Collection (Valencia, Spain) under the designator CECT11047, as well as the histidine-auxotrophic strain *P. pastoris* CBS7435 (Δ*HIS4*) from the Austrian Center of Industrial Biotechnology (Graz, Austria). *S. cerevisiae* INV*Sc*1 (genotype: *MATa HIS3*D1 *LEU2 TRP1*-289 *URA3*-52 *MAT HIS3*D1 *LEU2 TRP1*-289 *URA3*-52) from Invitrogen (Waltham, MA, USA) was used for *Saccharomyces* experiments. Shake flask cultivations of both *P. pastoris* and *S. cerevisiae* were performed using YPD (Yeast Peptone Dextrose) medium, as well as BMD (Buffered Minimal Dextrose) medium (Invitrogen, 2010) for *P. pastoris* and SC minimal medium (Invitrogen, 2008) for *S. cerevisiae*. If necessary, BMD medium was supplemented with 4 mg/L L-histidine, and SC medium was supplemented with 0.05 g/L L-histidine, 0.1 g/L L-leucine, and 0.1 g/L L-tryptophan. *P. pastoris* expression levels were assayed in 96-deep-well plates with 2.4 mL total volume (Eppendorf, Hamburg, Germany) according to Weis et al. (2004) and Hartner et al. (2008), using BMD, BMM2 (Buffered Minimal Methanol) and BMM10 medium. By applying this method, the cells are first grown in BMD and expression is induced with BMM2 and BMM10 by maintaining a 0.5% (*v/v*) methanol concentration in the culture medium. All yeast cultivations were carried out at 28°C, with shake flasks (baffled) being agitated at 120 min^-1^ and deep-well plates at 340 min^-1^.

### Genome Sequencing and Bioinformatic Analysis

Genomic DNA (gDNA) was isolated from yeast cultures using the MasterPure^TM^ Yeast DNA Purification kit (Epicentre, Madison, WI, USA). The method used for genome sequencing of relevant *P. pastoris* strains was recently described (Schwarzhans et al., 2016a). In short, gDNA quality was assayed via gel-electrophoresis and gDNA of sufficient quality was quantified using the Quant-iT PicoGreen dsDNA kit by Invitrogen (Waltham, MA, USA). From samples of high quality and quantity, paired-end libraries were prepared by applying the TruSeq sample preparation kit (Illumina, San Diego, CA, USA). The libraries were sequenced on an Illumina MiSeq system. Raw data was *de novo* assembled using the GS *De Novo* Assembler (Version 2.8, Roche, Basel, Switzerland) with default settings. The assembled draft genome of *P. pastoris* JPS664 can be found under FBUC01000000, and the finalized sequence of pMito under LT724168 in the EBI database.

For bioinformatic analysis the BLASTn algorithm (Altschul et al., 1997) and a local database including the pAHBgl-GFP vector sequence were used for database comparison. Only hits with a sequence identity of 100% and an e-value > 1 × 10^-20^ were further analyzed in more detail. If necessary, gaps in the vector were closed via an *in silico* approach with CONSED (Gordon et al., 1998; Küberl et al., 2011; Wibberg et al., 2011). This approach allowed to determine the exact locus for the expression cassette in the sequenced *P. pastoris* genome.

### Construction and Transformation of Vectors

Primers were designed in SnapGene (GSL Biotech, Chicago, IL, USA) and their sequences can be found in Supplementary Table S3. PCR procedures employed the Phusion^®^ High-Fidelity DNA Polymerase (New England Biolabs, Ipswich, MA, USA). For agarose gel electrophoresis analysis, the GeneRuler DNA Ladder Mix by Thermo Scientific (Waltham, MA, USA) was used as size marker. The *P. pastoris* strain JPS664 was created as described in Schwarzhans et al. (2016a) with a *Bgl*II digested pAHBgl-GFP plasmid. pMito and its variants were amplified via PCR using the original pMito as template. Potentially due to secondary structures, the variants pMito-ΔUTR and pMito-ΔGOI consistently resisted PCR amplification. As a compromise, primers were designed that bind slightly inside the region targeted for omission. In consequence, pMito-ΔUTR and pMito-ΔGOI still contain 63 and 65 bp of the targeted region, respectively. pYES2 was obtained from Invitrogen (Waltham, MA, USA). It can be used for the transformation of *S. cerevisiae* with an episomal vector containing a 2 μm sequence for replication and an *URA*3 selection marker (Invitrogen, 2008). For the construction of pYES2-Mito the mtDNA fragment on pMito was PCR amplified and combined via Gibson Assembly (Gibson et al., 2009) with the linearized pYES2 without 2 μm sequence.

*Pichia pastoris* CBS7435 (Δ*HIS*4) was transformed according to Wu and Letchworth (2004) and *S. cerevisiae* INV*Sc*1 according to Thompson et al. (1998) using PCR amplified, linear DNA. Per transformation approximately 500 ng of purified (Wizard^®^ Plus SV Minipreps DNA Purification System, Promega, Madison, WI, USA) DNA was used. After transformation the *Pichia* cells were immediately spread onto MD (Minimal Dextrose) plates (Invitrogen, 2010), and *Saccharomyces* cells onto SC plates without uracil (Invitrogen, 2008) in 200 μL aliquots and incubated for 3 days at 28°C. Following the incubation, the total number of transformants was counted and randomly selected clones picked for dilution plating. Single colonies from dilution plating were used for following experiments, including PCR assays for the correct construct, expression screenings and GCN determination. Experiments for assaying the transformation efficiency were carried out in biological triplicates.

### *In Silico* Analysis of pMito ARS

The mtDNA fragment on pMito was scanned for ACS motifs via the FIMO tool (Grant et al., 2011) of the MEME suite (Bailey et al., 2009). Both the 11 and 17 bp ACS motifs of *S. cerevisiae* (Newlon and Theis, 1993; Theis and Newlon, 1997), as well as the GC-ACS and AT-ACS motifs of *P. pastoris* (Liachko et al., 2014) were used as references.

### Plasmid Stability Assay

Precultures of *P. pastoris* JPS664 were grown overnight under selective conditions in MD medium without L-histidine. On the following day the main cultures were inoculated to OD 0.2. For selective conditions MD medium without L-histidine and for non-selective conditions YPD medium were used, respectively. Samples for OD measurement and the plasmid stability assay were taken every 2 h until 16 h of cultivation and again after 26, 36, and 60 h. OD values were used to calculate the specific growth rate at each time point. Before plating onto YPD plates, the samples for the plasmid stability assay were diluted based on the OD so that about 100–1000 colonies were to be expected per plate (Invitrogen, 2010). After 2 days of incubation, 52 colonies were picked per sampling point and individually washed twice with 200 μL of 9 g/L NaCl. Washed cells were resuspended in 20 μL of 9 g/L NaCl and pipetted onto MD plates without L-histidine. Following 2–3 days of incubation the colonies were counted in order to determine the plasmid stability. The plasmid stability assay was carried out in biological duplicates with technical triplicates each.

### Determination of GFP Expression Level and Gene Copy Number

The procedures employed for assaying the GFP expression level and GCN were described previously in more detail (Schwarzhans et al., 2016a). In brief, a reference strain containing a single copy of the GFP expression cassette in the *AOX1* locus was used for normalization of the GFP/OD expression level. Strains were grown in 96 deep-well plates as described above and values 60 h after the start of the methanol induction are presented in this study. All strains were cultivated in biological triplicates with technical triplicates each. The GCN was determined based on the protocol by Abad et al. (2010) via the 2^-ΔΔCt^ method (Livak and Schmittgen, 2001), with *ARG4* as the calibrator gene. Biological triplicates with technical duplicates each were used in these procedures.

### Super-Resolution Fluorescence Microscopy

Mitochondria were stained with MitoTracker^®^ Red CM-H_2_XRos (Thermo Scientific, Waltham, MA, USA). The staining procedure was performed as described by Farre et al. (2007), using *P. pastoris* cells that had been induced with 0.5% (*v/v*) methanol for 3 days. Per experiment 3 μL of cells were mounted between a microscope slide and a high precision #1.5 coverglass (Marienfeld-Superior, Germany). Images were acquired on a DeltaVision OMX V4 system from GE Healthcare (United Kingdom) with a 60x 1.42 NA oil immersion PlanApoN objective (Olympus, Japan) and sCMOS camera. This setup applies the principal of three dimensional super-resolved structured illumination microscopy (3D SR-SIM), gaining a twofold resolution increase compared to conventional fluorescence microscopy. GFPuv was excited at 488 nm and the emission recorded at 504–552 nm. For MitoTracker^®^ Red CM-H_2_-XRos an excitation wavelength of 568 nm and emission wavelength band of 590–627 nm were employed. Multiple z planes encompassing the entire cell from top to bottom were recorded at a distance of 125 nm. For reference, differential interference contrast (DIC) images were recorded. Super-resolved fluorescent images were reconstructed with the corresponding recorded optical transfer function (OTF) in the softWoRx 6.5.2 software (GE Healthcare, United Kingdom) at a Wiener filter setting of 0.006. The GFP background was adjusted by subtracting the value of the WT, non-expressing strain. Maximum intensities were individually adjusted for optimal representation. The raw data, OTFs and calibration settings can be provided upon request.

## Author Contributions

J-PS, JK, and KF designed, analyzed and interpreted wet lab experiments. J-PS and TL performed wet lab experiments. AW performed genome sequencing work. DW analyzed and interpreted sequencing data. WH carried out fluorescence microscopy experiments. J-PS, DW, and WH wrote the manuscript. TH, JK, and KF revised the manuscript. J-PS, JK, and KF conceived the study. TH, JK, and KF supervised the research. All authors read an approved the final manuscript.

## Conflict of Interest Statement

The authors declare that the research was conducted in the absence of any commercial or financial relationships that could be construed as a potential conflict of interest. The reviewer FG and handling Editor declared their shared affiliation, and the handling Editor states that the process nevertheless met the standards of a fair and objective review.
