# Supplementary material for: A Mitochondrial Autonomously Replicating Sequence from Pichia pastoris for Uniform High Level Recombinant Protein Production
Source: Front Microbiol. 2017 May 2;8:780. doi: 10.3389/fmicb.2017.00780 (PMC5411459; doi:10.3389/fmicb.2017.00780)
Supplement: Supplementary file 1 [file Data_Sheet_1.DOCX]

Supplementary Material

A Mitochondrial Autonomously Replicating Sequence from *Pichia pastoris* for Uniform High Level Recombinant Protein Production

Jan-Philipp Schwarzhans, Daniel Wibberg, Anika Winkler, Tobias Luttermann, Wolfgang Hübner, Thomas Huser, Jörn Kalinowski, Karl Friehs^*^

*** Correspondence:** Karl Friehs: karl.friehs@uni-bielefeld.de

**
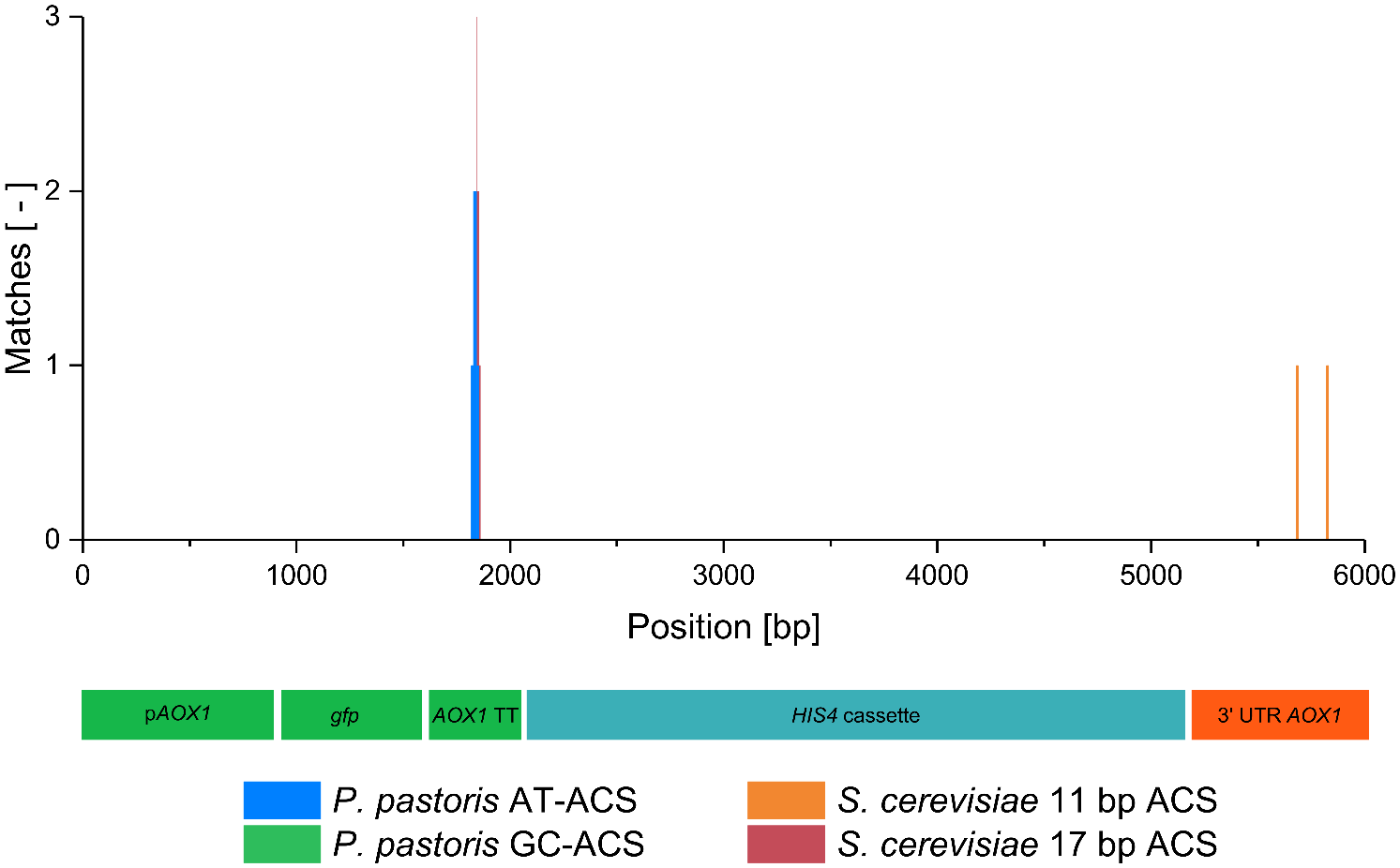
**

**Figure S1:** Cumulative matches of the FIMO scan for *P. pastoris* AT- and GC-ACS, as well as *S. cerevisiae* 11 and 17 bp ACS motifs mapped to the expression cassette segment of pMito, excluding the mtDNA fragment. The regions of the different features on the expression cassette are shown below the x-axis.

**Table S1**: FIMO matches of the AT-ACS and GT-ACS from *P. pastoris*, as well as the 11 and 17 bp ACS from *S. cerevisiae* to the 1442 bp mtDNA fragment in pMito.

| **Motif** | **Strand** | **Start** | **End** | **p-value** | **q-value** | **Matched Sequence** |
| --- | --- | --- | --- | --- | --- | --- |
| ***P. pastoris* AT-ACS** | + | 1260 | 1286 | 1.51e-06 | 0.000891 | AAATATAAACTTAAAAATATAGTTTCC |
|  | + | 702 | 728 | 3.38e-06 | 0.000891 | TTATGTATTTTTATTTCTATACTTTTT |
|  | + | 32 | 58 | 3.61e-06 | 0.000891 | TGATACAAATAATACTTTATATCATTT |
|  | + | 387 | 413 | 4.52e-06 | 0.000891 | AATTCTAAATTACATTCTATACGATTT |
|  | - | 1257 | 1283 | 7.67e-06 | 0.00121 | AACTATATTTTTAAGTTTATATTTACT |
|  | + | 657 | 683 | 1.1e-05 | 0.00144 | ATATGTAATTTATCTTGTATACGAGTT |
|  | + | 1043 | 1069 | 2.27e-05 | 0.00256 | TAACATAACAAGTTTTATATTGTTTTG |
|  | + | 1057 | 1083 | 2.75e-05 | 0.00271 | TTATATTGTTTTGACTATTAATTTTAT |
|  | + | 149 | 175 | 5.9e-05 | 0.00435 | TTCTATAAATTTTAATATTTTTGATAT |
|  | - | 158 | 184 | 6.42e-05 | 0.00435 | TTTTATAGAATATCAAAAATATTAAAA |
|  | + | 1274 | 1300 | 6.45e-05 | 0.00435 | AAATATAGTTTCCAGCATATAGTCAAA |
|  | - | 130 | 156 | 6.63e-05 | 0.00435 | TTATAGAAATACGGAAATATGCCAATT |
|  | + | 723 | 749 | 7.33e-05 | 0.00436 | CTTTTTAATTTATTTTTTCCATTTATT |
|  | + | 714 | 740 | 7.74e-05 | 0.00436 | ATTTCTATACTTTTTAATTTATTTTTT |
|  | + | 837 | 863 | 9.6e-05 | 0.00505 | TTTAATAATTCTATTTTTGTATTTCTC |
| ***P. pastoris* GC-ACS** | + | 928 | 947 | 1.96e-06 | 0.00558 | CGGGGATCGAACCTAGTCCG |
| ***S. cerevisiae* 11 bp ACS** | + | 159 | 169 | 1.01e-05 | 0.00461 | TTTAATATTTT |
|  | - | 1259 | 1269 | 1.47e-05 | 0.00461 | GTTTATATTTA |
|  | + | 998 | 1008 | 1.61e-05 | 0.00461 | TTTTAAGTTTT |
|  | + | 730 | 740 | 2.66e-05 | 0.00461 | ATTTATTTTTT |
|  | + | 743 | 753 | 2.66e-05 | 0.00461 | ATTTATTTTTT |
|  | + | 376 | 386 | 2.82e-05 | 0.00461 | TATTATATTTT |
|  | - | 1265 | 1275 | 4.01e-05 | 0.00522 | TTTTAAGTTTA |
|  | + | 914 | 924 | 4.25e-05 | 0.00522 | ATCTATATTTT |
|  | + | 350 | 360 | 7.79e-05 | 0.00768 | ATTTAAATTTA |
|  | + | 812 | 822 | 8.39e-05 | 0.00768 | ATCTATATTTA |
|  | + | 798 | 808 | 8.6e-05 | 0.00768 | TTTAAAATTTT |
| ***S. cerevisiae* 17 bp ACS** | + | 347 | 363 | 5.2e-06 | 0.00433 | ATTATTTAAATTTAATT |
|  | + | 734 | 750 | 1.31e-05 | 0.00543 | ATTTTTTCCATTTATTT |
|  | - | 1262 | 1278 | 6.04e-05 | 0.0106 | TATTTTTAAGTTTATAT |
|  | - | 1256 | 1272 | 7.21e-05 | 0.0106 | TAAGTTTATATTTACTA |
|  | + | 1052 | 1068 | 7.34e-05 | 0.0106 | AAGTTTTATATTGTTTT |
|  | + | 701 | 717 | 7.66e-05 | 0.0106 | ATTATGTATTTTTATTT |

**Table S2**: FIMO matches of the AT-ACS and GT-ACS from *P. pastoris*, as well as the 11 and 17 bp ACS from *S. cerevisiae* to the 5884 bp expression cassette region of pMito, excluding the mtDNA fragment.

| **Motif** | **Strand** | **Start** | **End** | **p-value** | **q-value** | **Matched Sequence** |
| --- | --- | --- | --- | --- | --- | --- |
| ***P. pastoris* AT-ACS** | + | 1818 | 1844 | 9.25e-05 | 0.487 | ATTTGTAACCTATATAGTATAGGATTT |
|  | - | 1829 | 1855 | 9.33e-05 | 0.487 | AATGACAAAAAAAATCCTATACTATAT |
| ***S. cerevisiae* 11 bp ACS** | - | 5820 | 5830 | 4.14e-05 | 0.276 | TTTCACGTTTA |
|  | - | 5680 | 5690 | 5.03e-05 | 0.276 | TTTAACGTTTA |
| ***S. cerevisiae* 17 bp ACS** | + | 1844 | 1860 | 9.85e-06 | 0.106 | TTTTTTGTCATTTTGTT |

**Table S3**: Names, nucleotide sequences and purposes of all primers used in this study.

| **Name** | **Sequence** | **Purpose** |
| --- | --- | --- |
| pMito_Circ_mtDNA-FW | GTCCTTCGTACTATTTAAAGAGAAC | Amplification of pMito and pYES2-Mito, linearized inside mtDNA fragment |
| pMito_Circ_mtDNA-RV | GAATTAAGTTCGACCTATCGGAG |  |
| pMito_Circ_HIS4-FW | GAATTGGATTGTGATGGAGACTGC | Amplification of pMito, linearized inside *HIS4* |
| pMito_Circ_HIS4-RV | GATACCCAGCAACTTTTGAGTTGC |  |
| pMito_No_GOI-FW | GGAACGGAACGTATCTTAGCATGGT | Amplification of pMito-ΔGOI from pMito |
| pMito_No_GOI-RV | TGGACCTGTGGATGTCGGATGG |  |
| pMito_No_UTR-FW | TGCGGAGATGCGATATCGAG | Amplification of pMito-ΔUTR from pMito |
| pMito_No_UTR-RV | AATGCGAGGATGCTGCTGG |  |
| pYES2_Circ-FW | CCTAATATCCGACAAACTGTTTTACAG | Amplification of pYES2, linearized ca. 300 bp upstream of 2 µm region |
| pYES2_Circ-RV | CTGTATCTCCTCAAAGCGTATTCG |  |
| mtDNA_Insert-FW | TCAATAGCATATCTTTGTTATATAGTGGACTATCTCTTCAATCTTAATTTTG | Amplification of mtDNA fragment from pMito for insertion via Gibson assembly into pYES2. Overlapping bases to pYES2 are underlined. |
| mtDNA_Insert-RV | ACCGGGGTGGAGCTTCCCAGGATTATCAGGGGTAGTATTATCAAATG |  |
| pYES2_No_2um-FW | GGGAAGCTCCACCCCGGTTG | Linearization of pYES2 without 2 µm region for Gibson assembly of pYES2-Mito |
| pYES2_No_2um-RV | AACAAAGATATGCTATTGAAGTGC |  |
